# RNAseq of TGF-β receptor type I kinase-dependent genes in oral fibroblast exposed to milk

**DOI:** 10.1186/s12903-021-01913-5

**Published:** 2021-11-16

**Authors:** Layla Panahipour, Dariush Mehdipour Moghaddam, Jila Nasirzade, Zahra Kargarpour, Reinhard Gruber

**Affiliations:** 1 Department of Oral Biology, Medical University of Vienna, Sensengasse 2a, 1090 Vienna, Austria; 2 Department of Periodontology, School of Dental Medicine, University of Bern, Freiburgstrasse 7, 3010 Bern, Switzerland; 3 Austrian Cluster for Tissue Regeneration, Donaueschingenstraße 13, 1200 Vienna, Austria

**Keywords:** Human milk, TGF-β activity, TGF-β receptor type I kinase, Gingival fibroblast, In vitro

## Abstract

**Background:**

Milk is a rich source of natural growth factors that may support oral tissue homeostasis and wound healing. We had shown earlier that blocking TGF-β receptor type I kinase with the inhibitor SB431542 abolished the expression of IL11 and other genes in human gingival fibroblasts exposed to the aqueous fraction of milk. Our aim was to identify the entire signature of TGF-β receptor type I kinase-dependent genes regulated by the aqueous fraction of human milk.

**Result:**

RNAseq revealed 99 genes being strongly regulated by milk requiring activation of the SB431542-dependent TGF-β receptor type I kinase. Among the SB431542-dependent genes is IL11 but also cadherins, claudins, collagens, potassium channels, keratins, solute carrier family proteins, transcription factors, transmembrane proteins, tumor necrosis factor ligand superfamily members, and tetraspanin family members. When focusing on our candidate gene, we could identify D609 to suppress IL11 expression, independent of phospholipase C, sphinosine-1 phosphate synthesis, and Smad-3 phosphorylation and its nuclear translocation. In contrast, genistein and blocking phosphoinositide 3-kinases by wortmannin and LY294002 increased the milk-induced IL11 expression in gingival fibroblasts.

**Conclusion:**

Taken together, our data revealed TGF-β receptor type I kinase signaling to cause major changes of the genetic signature of gingival fibroblasts exposed to aqueous fraction of human milk.

**Supplementary Information:**

The online version contains supplementary material available at 10.1186/s12903-021-01913-5.

## Introduction

Milk being the hallmark of mammalian evolution contains the entire spectrum of nutrients that newborns require for their growth and development [1]. Apart from the nutritional aspects, milk holds a broad spectrum of biological active molecules exerting various beneficial activities including the reduction of inflammation and supporting oral tolerance [2, 3]. The biological active molecules can be roughly distinguished into proteins, lipids and carbohydrates [4]. Among the bioactive proteins are growth factors, interferon [5], chemokines [6] and cytokines. Milk is also a rich source of antimicrobial proteins and peptides, such as lactoferrin, defensins, and cathelicidins [7], as well as α-lactalbumin, β-lactoglobulin, lactoferrin, osteopontin, immunoglobulins, lactoperoxidase and other enzymes [8]. The contemporary proteomic approach revealed the milk protein signature [9]. The complex lipids include sphingolipids, such as sphingomyelin and gangliosides [10]. The human milk oligosaccharides are exemplified by 2'-fucosyllactose [11]. Owing to this large spectrum of molecules, the cellular response to milk is manifold. It is not clear to which extend each of the various molecules is responsible for a certain cellular effect.

The most abundant growth factors in milk and colostrum are transforming growth factor (TGF)-β2 and TGF-β1, insulin-like growth factors, epidermal growth factors, and basic fibroblast growth factor that can be identified by immunoassay and bioassay [12]. Activin A being part of the TGF-β superfamily and its binding protein follistatin were also identified in human milk [13]. To detect the TGF-β activity of milk and dairy products, cells responding to TGF-β by an increased expression of target genes can be used [14–16]. Among these target genes is IL11 being strongly increased in fibroblasts exposed to aqueous fraction of milk and related products [14–16]. Further support for the TGF-β activity of milk is provided by the TGF-β receptor type I kinase inhibitor SB431542 [17, 18]. In this setting, SB431542 abolished the potential of milk to increase the expression of IL11 [14, 15]. The TGF-β receptor type I kinase inhibitor SB431542 also helped to confirm the TGF-β activity in enamel matrix derivatives [19], bone lysates and conditioned medium [20, 21], and platelet-rich fibrin [22, 23]. However, to which extend the signature of milk-induced genes, including IL11, depends on the TGF-β receptor type I kinase remains to be determined.

RNA sequencing (RNAseq) is a recently developed approach to transcriptome profiling that uses deep-sequencing technologies [24]. Transcriptome profiling was applied to identify the SB431542-dependent genes being strongly regulated by enamel matrix derivatives in oral fibroblasts [19]. Here we follow this approach with our aim to identify the TGF-β receptor type I kinase-dependent genes such as IL11 to be regulated by the aqueous fraction of human milk in gingival fibroblast. IL11 is not simply a strongly-regulated target gene as it is critically involved in mediating downstream TGF-β effects in cardiovascular and liver fibrosis [25, 26]. We also have shown previously that milk and its products cause the activation of the canonical TGF-β signaling pathway that culminates in the phosphorylation of Smad-3 and nuclear translocation of Smad2/3 [14–16]. However, that does not necessity mean that all TGF-β receptor type I kinase-dependent genes are driven by the canonical signaling or are expressed in consequence of downstream metabolites such as sphingosine 1-phosphate (S1P) [27] being critical for TGF-β-stimulated tissue fibrosis [28]. It thus requires a basal approach to identify possible second messenger signaling pathways that drive the expression of target genes such as IL11 downstream or even independent of TGF-β receptor type I kinase activation in gingival fibroblasts.

## Results

### RNA sequencing of gingival fibroblasts exposed to milk

**Fig. 1:**
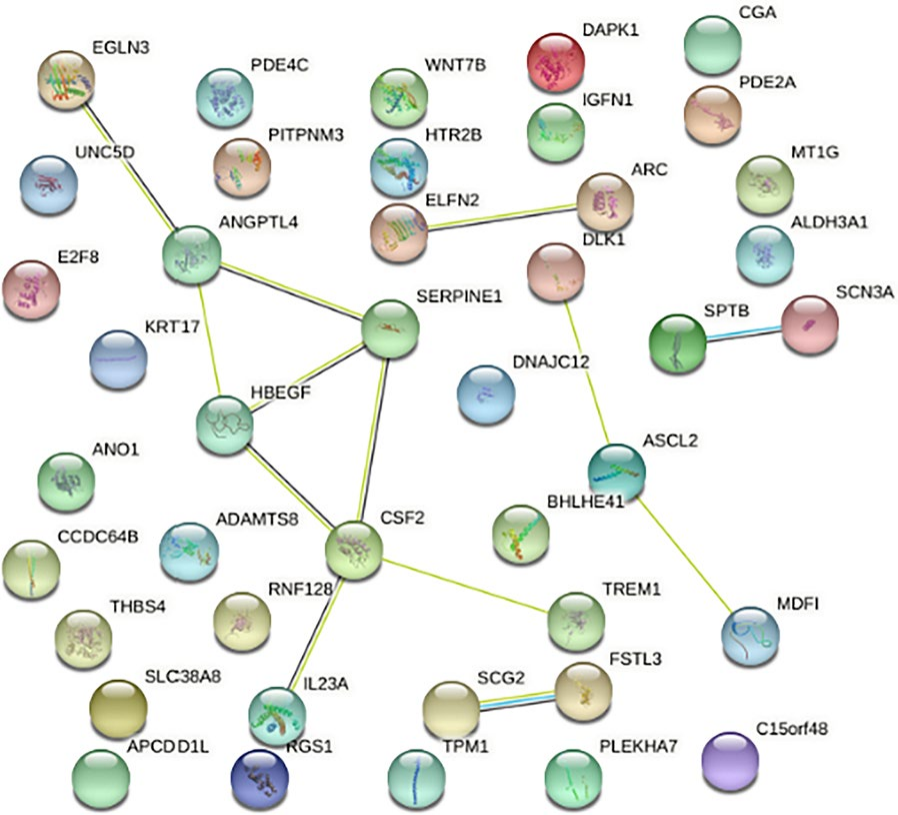
STRING network of non-TGF‐β RI kinase regulated genes in gingival fibroblasts exposed to milk. Gingival fibroblasts were exposed to the aqueous fraction of 5% human milk with and without SB431542 followed by RNAseq. This STRING network represents the genes that are at least ninefold regulated by milk but independent of SB431542

To identify the most strongly regulated genes in gingival fibroblasts exposed to 5% of the aqueous fraction of human milk, an RNA sequencing approach was conducted. RNA sequencing revealed a total number of 142 genes being at least ninefold up- and down-regulated by milk (Additional file 1: Table S1). Among all milk-regulated genes, 70%, that are 99 genes, were blocked by SB431542, the inhibitor for the TGF-β receptor type I kinase. That means, only 43 genes were regulated by milk but independent of SB431542-dependent TGF-β receptor type I kinase signaling (Fig. 1), the latter being enriched for GO:0010469 (regulation of signaling receptor activity 10/577, ARC, CGA, CSF2, DAPK1, HBEGF, IL23A, SCG2, SERPINE1, THBS4, WNT7B) and GO:0044421 (extracellular region part 13/1375 ADAMTS8, ANGPTL4, ARC, CGA, CSF2, DLK1, FSTL3, HBEGF, IL23A, SCG2, SERPINE1, THBS4, WNT7B).

### Network analysis of SB431542-dependent genes being regulated in gingival fibroblasts exposed to milk

**Fig. 2:**
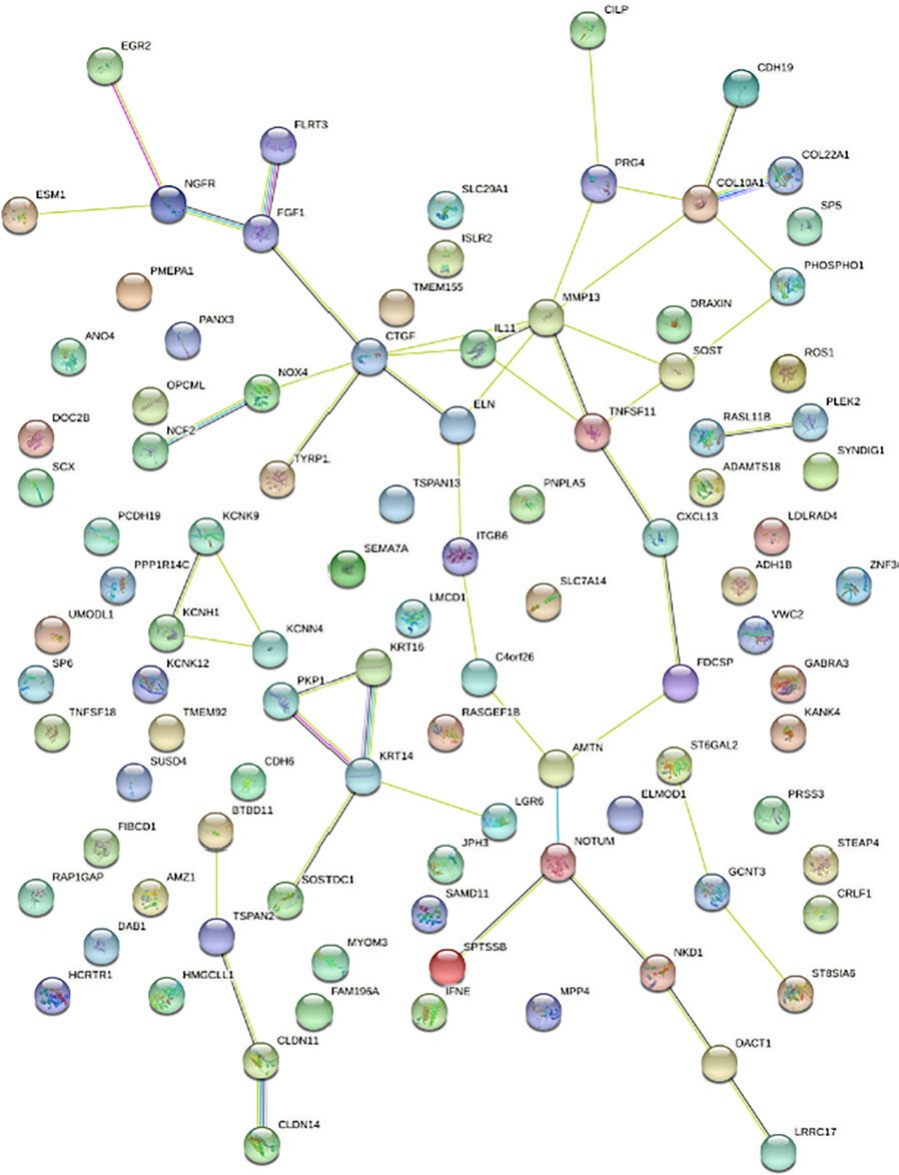
STRING network of TGF‐β RI kinase regulated genes in gingival fibroblasts exposed to milk. Gingival fibroblasts were exposed to the aqueous fraction of 5% human milk with and without SB431542 followed by RNAseq. This STRING network represents the genes that are at least ninefold regulated by milk being significantly blocked by SB431542

**Fig. 3:**
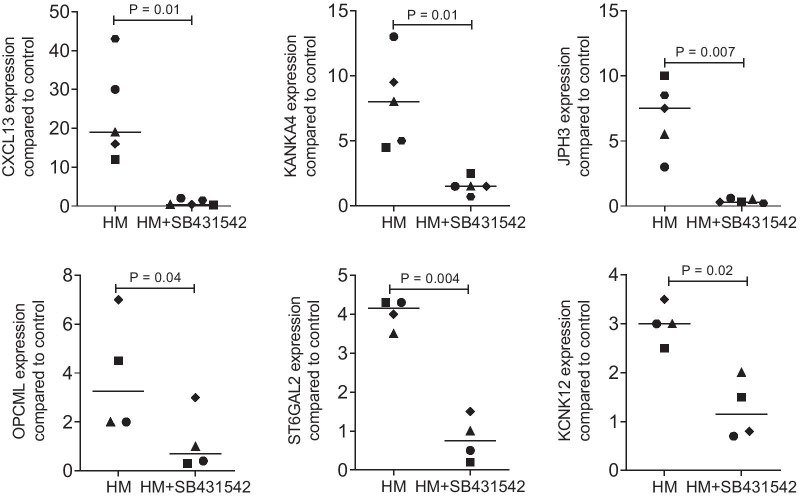
RT-PCR regulated genes in gingival fibroblasts exposed to milk. Gingival fibroblasts were exposed to 5% of an aqueous fraction of human milk (HM) with and without 10 µM SB431542 followed by RT-PCR. Statistical analysis of independent experiments was based on a paired t-test

Among the 99 genes requiring TGF-β receptor type I kinase signaling (Fig. 2) include (1) claudins (CLDN11, CLDN14); (2) keratins (KRT14, KRT16); (3) potassium channels (KCNH1, KCNK9, KCNN4), and (4) collagens (COL10A1, COL22A1), as well as other genes such as FGF1, NGFR, EGR2, NOX4, CTGF, IL11, MMP13, SOST, and TNFSF11. To further confirm some of the RNAseq data we have performed RT-PCR analysis of a panel of genes, namely CXCL13, KANKA4, JPH3, OPCML, ST6GAL2, and KCNK12 (Fig. 3). As expected from our previous research with IL11, NOX4 and PRG4 [14–16], SB431542 blocked the milk-dependent increase of gene expression in gingival fibroblasts (Fig. 3) and similarly in fibroblasts isolated from the periodontal ligament (data not shown). The oral squamous cell carcinoma cell HSC2, when exposed to milk, increased ID1 and ID3 genes [29] but failed to increase IL11 (data not shown). Taken together, the SB431542-dependent genes regulated by milk control a large spectrum of cellular responses that include but are not limited to the expression of paracrine signaling molecules.

### SB431542 and LY2157299 but not SIS3 block IL11 in gingival fibroblasts exposed to milk

**Fig. 4:**
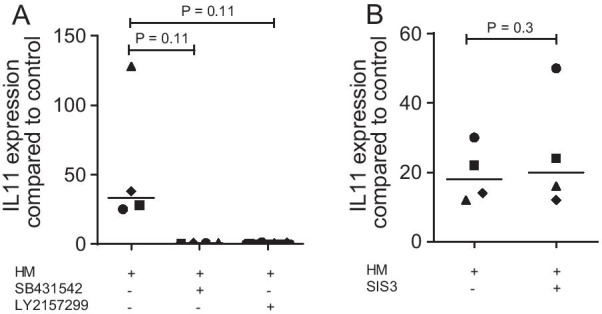
SB431542 and LY2157299 but not SIS3 block IL11 in gingival fibroblasts exposed to milk. Gingival fibroblasts were exposed to 5% of an aqueous fraction of human milk (HM) with and without 10 µM SB431542, 10 µM LY2157299, and 50 µM SIS3 followed by RT-PCR. Statistical analysis of four independent experiments was based on a paired t-test

Recently, we have shown that SB431542 blocks IL11 being strongly up-regulated by the aqueous fraction of milk and its products [14–16]. We now have included another inhibitor of TGF-β receptor type I kinase signaling, namely LY2157299, again confirming that the expression of IL11 induced by human milk strictly depends on TGF-β receptor type I kinase signaling (Fig. 4A). Considering that the canonical TGF-β signaling pathway requires Smad2/3 activation, SIS3, the Smad-3 inhibitor was introduced [30]. SIS3, surprisingly, failed to reduce the milk-induced expression of IL11 in gingival fibroblasts (Fig. 4B), which led us to reach out for alternative inhibitors of TGF-β signaling. Moreover, follistatin, a potent antagonist of activin neutralizing its activity, did not lower the capacity of milk to stimulate the expression of IL11 in gingival fibroblasts (data not shown).

### D609 blocks IL11 in gingival fibroblasts exposed to milk

**Fig. 5:**
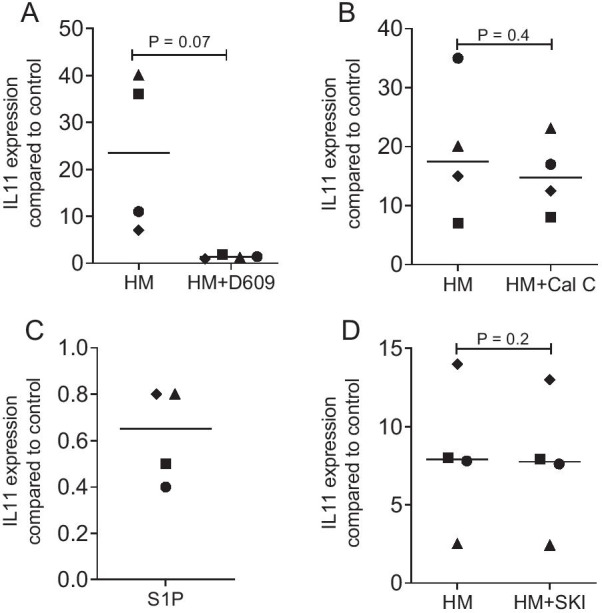
D609 blocks IL11 in gingival fibroblasts exposed to milk. Gingival fibroblasts were exposed to 5% of an aqueous fraction of human milk (HM) with and without 40 µM D609 (**A**), an aqueous fraction of human milk (HM) with and without 200 nM calphostin C (**B**), sphingosine 1-phosphate 50 µM (**C**) an aqueous fraction of human milk (HM) with and without 10 µM sphingosine kinase 1 inhibitor (**D**) followed by RT-PCR. Statistical analysis of four independent experiments was based on a paired t-test

We next introduced D609, a classical phosphatidylcholine-phospholipase C (PLC) inhibitor that blocked TGF-β-induced expression of fibronectin in fibroblasts [31] and a transgene in A549 cells [32] to our assay. Indeed, 40 µM D609 even abolished the milk-induced expression of IL11 in gingival fibroblasts (Fig. 5A). However, blocking a downstream mediator of PLC, i.e. PKC with 200 nM calphostin C failed to reduce the IL11 expression induced by milk (Fig. 5B). D609 is not specific for PLC as it also inhibits sphingomyelin synthesis [33] and TGF-β can increase sphingomyelin in synoviocytes [34]. However, neither exposing gingival fibroblasts to sphingosine 1-phosphate alone (Fig. 5C) nor blocking of sphingosine kinase 1 with SKI II (Fig. 5D) changed the expression of IL11.

### Smad-3 Western blot and immunostaining of gingival fibroblasts exposed to milk

**Fig. 6:**
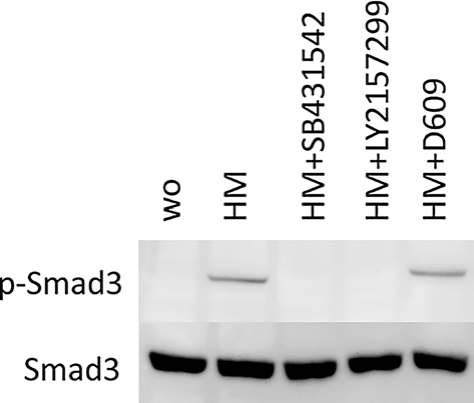
Smad-3 Western of gingival fibroblasts exposed to milk. Serum-starved gingival fibroblasts were exposed to 5% of an aqueous fraction of human milk (HM) with and without 10 µM SB431542, 10 µM LY2157299 and 40 µM D609 for 30 min before being subjected to Western blot analysis of phosphorylation of Smad-3. Cells exposed to human milk (HM) caused a strong increase in the phosphorylation of Smad-3 which was blocked by SB431542, LY2157299 but not by D609. ”wo” stands for without, indicating the unstimulated cells

**Fig. 7:**
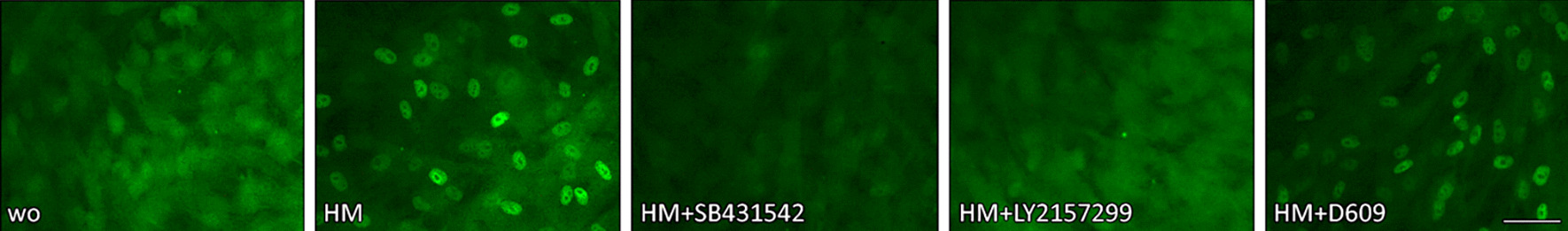
Smad2/3 immunostaining of gingival fibroblasts exposed to milk. Serum-starved gingival fibroblasts were exposed to 5% of an aqueous fraction of human milk (HM) with and without 10 µM SB431542, 10 μM LY2157299 and 40 µM D609 for 30 min before fluorescent labelling of Smad2/3. The nuclear signal is visible with cells exposed to human milk (HM) and blocked by SB431542, LY2157299 but not by D609. The characteristic nuclear signal is indicated by demarcation of the relatively stronger green color towards the faintly stained cytoplasm. Scale bar indicates 100 µm. ”wo” stands for without, indicating the unstimulated cells

To study if D609 affects the canonical TGF-β signaling, the phosphorylation of Smad-3 was visualized by Western blot analysis. Consistent with the critical involvement of TGF-β receptor type I kinase signaling, SB431542 and LY2157299 completely blocked the milk-induced phosphorylation of Smad-3 (Fig. 6) but D609 had no effect on the phosphorylation of Smad-3 (Fig. 6). These findings suggest that the D609-induced reduction of IL11 expression does not depend on phosphorylation status of Smad-3. Further support for this suggestion comes from nuclear translocation analysis, where again, SB431542 and LY2157299 completely blocked the milk-induced translocation of Smad2/3, while D609 was without any effects (Fig. 7). Taken together, the data point towards a milk-induced TGF-β receptor type I kinase signaling that causes the phosphorylation of Smad-3, which in turn drives the D609-dependent IL11 expression.

### Genistein, LY294002 and wortmannin increased IL11 in gingival fibroblasts exposed to milk

**Fig. 8:**
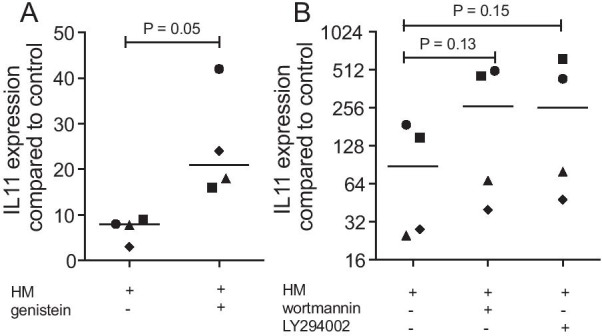
Genistein, LY294002 and wortmannin increased IL11 in gingival fibroblasts exposed to milk. Gingival fibroblasts were exposed to 5% of an aqueous fraction of human milk (HM) with and without (A) 40 µM genistein, and (B) 100 µM wortmannin, and 10 µM LY294002 followed by RT-PCR. Statistical analysis of four independent experiments was based on a paired t-test

Finally, we performed a screening for the possible involvement of protein tyrosine kinase and phosphoinositide 3-kinases (PI3K) signaling to modulate the effects of milk on IL11 expression in gingival fibroblasts. Genistein, a phytoestrogen of soy products, being a potent inhibitor of PTK (Fig. 8A), and also LY294002 and wortmannin that act as potent inhibitors of phosphoinositide 3-kinases (PI3Ks; Fig. 8B), enhance the already strong expression of IL11 in response to milk.

## Discussion

This study was inspired by our observations that milk and dairy products not only are rich sources of TGF‐β but also cause a robust increase of IL11 and other genes via activation of TGF-β receptor type I kinase on gingival fibroblasts [14–16]. Milk, however, is a rich cocktail of bioactive molecules including growth factors other than TGF‐β as well as lipids and oligosaccharides [4–11]. The first question arising is to which extend the response of gingival fibroblasts to milk depends on the TGF-β receptor type I kinase? We have previously reported that inhibiting the TGF-β receptor type I kinase with SB431542 blocked the entire spectrum of strongly regulated genes in gingival fibroblasts exposed to enamel matrix derivative [19], a crude extract of porcine fetal tooth material [35]. This research was in support of the TGF-β activity of enamel matrix derivative [36, 37]. The same research strategy was now performed to understand to which extent the TGF-β activity of human milk accounts responsible for the gene expression changes in gingival fibroblasts. Another aim was to identify the signaling mechanisms downstream of TGF-β receptor type I kinase focusing on the expression of the strongly regulated IL11.

The major finding according to our first aim was in line with what we have learned from using enamel matrix derivative [19] that the majority of strongly regulated genes in gingival fibroblasts by milk require the activity of the TGF-β receptor type I kinase. Support for this claim comes from comparing the SB431542-dependent transcriptomes of enamel matrix derivative and milk that include IL11 [19, 38] but also CILP, CXCL13, ECR2, KANK4, LMCD1, NCF2, NOX4, PLEK2, PRG4, PMEPA1, SCX, SEMA7A, TMEM26, and ZNF365. Thus, there is a similar change of the transcriptome when fibroblasts are exposed to enamel matrix derivative and milk. A comparable research strategy with HTS466284 [39] was used to identify the role of TGF-β receptor type I kinase in mediating the anti-inflammatory activity of milk in a mouse colitis model [40]. There is thus good evidence that, even though milk holds a large spectrum of bioactive components, it is the TGF‐β activity that has a major impact on the gingival fibroblast target cells. In line with this claim, follistatin, an activin neutralizing protein failed to modulate the milk-induced IL11 expression.

Even though not in the focus of this research is the transcriptome changed by milk independent of SB431542. For example, cytokines and growth factors such as CSF2, IL23A, WNT7A, and protease such as SERPINE1 are regulated by milk but independent of SB431542. Nevertheless, care should be taken when interpreting these findings because TGF‐β can increase CSF2 in uterine epithelial cells [41], WNT7A in chondrocytes [42], KRT17 in tumor epithelial cells [43], and SERPINE1 in renal epithelial cells [44]. TGF‐β can further reduce ADAMTS8 in alveolar epithelial cells [45]. Considering that TGF-β also signals directly via TGF-β receptor type II kinase, SB431542 does not abolish the overall TGF-β activity [46]. Moreover and considering that milk is a rich source of TGF-β2 having a different affinity for the TGF-β receptors than TGF-β1 and TGF-β3, an SB431542-independent activity should not be ruled out [47]. Our data should therefore be interpreted with respect to a possible SB431542-independent TGF-β activity observed with gingival fibroblasts.

The expression of SB431542-dependent genes is maybe not exclusively a consequence of canonical Smad-3 signaling pathway. Thus, one major finding according to this aim was not that milk causes the TGF-β receptor type I kinase dependent phosphorylation of Smad-3 and the nuclear translocation of Smad2/3 – it was that D609 blocked the milk-induced expression of IL11 in gingival fibroblasts. Consistent with this observation, D609 blocks TGF-β1-induced transcription of fibronectin in fibroblasts [31] and a transgene in A549 cells [32]. Considering that PLC activates PKC, we performed PKC blocking experiments but calphostin C did not reduce the IL11 expression induced by milk. Thus, the PLC-PKC pathways is not responsible for the changes in IL11 gene expression. D609 failed to reduce the canonical Smad-3 signaling pathway suggesting that D609 acts downstream of Smads.

D609 also inhibits the sphingomyelin synthase [33] and TGF‐β can increase sphingomyelin in synoviocytes [34]. We therefore asked if sphingomyelin syntheses and its products sphingomyelin and 1,2-diacyl-sn-glycerol regulate IL11 expression in fibroblasts. Support for this concept comes from the observation that S1P, a bioactive lysophospholipid originating from sphingomyelin, can induce IL11 in smooth muscle cells [27]. Moreover, S1P is critical for TGF-β-stimulated tissue fibrosis [28]. We thus proposed a D609-dependent activation of sphingosine kinase 1 (SphK1) expression that accompanied S1P production acting downstream of the canonical Smad2/3 signaling [48]. However, blocking sphingosine kinase 1 failed to modulate the milk-induced expression of IL11, and consistently, also S1P could not induce IL11 expression in oral fibroblasts. Our observation therefore suggests that milk-derived TGF‐β does not induce the IL11 expression via the activation of the sphingosine kinase/sphingosine-1-phosphate pathway.

Interestingly, however, genistein, a phytoestrogen found in soy products, with a highly specific inhibitor of protein tyrosine kinase (PTK), and also blocking of PI3K with wortmannin and LY294002 even increased the expression of IL11 in the presence of milk. These findings suggest that apart from milk-derived TGF-β inducing the expression of IL11, milk supposedly activated pathway including PTK and PI3K that lower the expression of IL11 in the gingival fibroblasts. Future research needs to identify the signaling pathways of how D609 blocks, and genistein and the two PI3k inhibitors wortmannin and LY294002 even push IL11 expression in our experimental setting.

In summary, we have identified the genetic signature of gingival fibroblasts exposed to the aqueous fraction of human milk while blocking the TGF-β receptor type I kinase by SB431542. We also demonstrate that D609 is a potent inhibitor of IL11 expression, and that genistein as well as the PI3K inhibitors can increase milk-induced IL11 expression in oral fibroblasts. This study remains descriptive, but may be a primer for future research do better understand the biological role of milk TGF-β activity for deciphering the overall impact of milk and dairy product on oral health during adulthood.

## Material and methods

### Human milk

Human milk samples were collected at the Department of Paediatric and Adolescent Medicine, Division of Neonatology, of the Medical University of Vienna after receiving an informed consent and the approval of the ethics committee of the Medical University of Vienna (1021/2017). Milk was prepared in daily totals for premature babies and delivered to the feeding stations. Three leftover residual amounts were used for the present study. No mother donated the milk for the study purpose by primary intension. All experiments were performed in accordance with relevant guidelines and regulations. Human milk was centrifuged at 20,000 *g* for 10 min. The aqueous fraction was harvested and stored frozen until further use.

### Gingival fibroblasts—basic experimental setting

Human gingiva was removed from extracted healthy wisdom teeth of patients who had signed an informed consent. The harvesting procedure was approved by Ethics committee of Medical University of Vienna (EK NR 631/2007), Vienna, Austria. All experiments were conducted in compliance with relevant guidelines and regulations. Experiments were performed by a pool of three different strains of fibroblasts derived from the explants, passaged less than 10 times. Cells were cultured in a humidified atmosphere at 37 °C, 5% CO_2_, and 95% humidity in growth medium consisting of DMEM, 10% fetal calf serum and 1% antibiotics (Invitrogen Corporation, Carlsbad, CA, USA). Cells were plated in growth medium at 30,000 cells/cm^2^ in to 6 well plates. The following day, cells were incubated with three individual preparations of 5% aqueous fraction of human milk in serum-free DMEM for 18 h. The inhibitor for the TGF-β RI kinase, SB431542 (Calbiochem, Merck, Billerica, MA, USA) and LY2157299 (Cayman Chemical, Ann Arbor, MI, USA) was used at 10 µM. D609 (Calbiochem, Merck, Billerica, MA, USA) and genistein (Sigma) were used at 40 µM. Sphingosine kinase inhibitor 2 blocking SPHK1 (SKI II; Cayman Chemical, Ann Arbor, MI, USA) and sphingosine 1-phosphate (Sigma, St. Louis, MO) was used at 10 µM and 100 µM, respectively. Cells were also exposed to 200 nM calphostin c, 100 µM wortmannin, and 10 µM LY294002 (all Cayman Chemical) in the presence of milk. In the same setting, follistatin was used at 200 ng/mL (Sigma, St. Louis, MO) and the Smad-3 inhibitor SIS3 at 50 µM working concentration (Merck, Billerica, MA, USA).

### RNA sequencing

Total RNA was extracted with the RNA Isolation Kit (Extractme, BLIRT S.A., Gdańsk, Poland). RNA quality was evaluated using the Agilent 2100 Bioanalyzer (Agilent Technologies, Santa Clara, CA, USA). Sequencing libraries were prepared at the Core Facility Genomics, Medical University of Vienna using the NEBNext Poly (A) mRNA Magnetic Isolation Module and the NEBNext Ultra™ II Directional RNA Library Prep Kit for Illumina according to manufacturer's protocols (New England Biolabs). Libraries were QC-checked on a Bioanalyzer 2100 (Agilent) using a High Sensitivity DNA Kit for correct insert size and quantified using Qubit dsDNA HS Assay (Invitrogen). Pooled libraries were sequenced on a NextSeq500 instrument (Illumina) in 1 × 75 bp single-end sequencing mode. Approximately 25 million reads were generated per sample. Reads in fastq format were aligned to the human reference genome version GRCh38 (www.ncbi.nlm.nih.gov/grc/human) with Gencode 29 annotations (www.gencodegenes.org/human/release_29.html) using STAR aligner 55 version 2.6.1a in 2-pass mode. Reads per gene were counted by STAR, and differential gene expression was calculated using DESeq2 56 version 1.22.2. DESeq2 results with a padj < 0.05 and a log2fc of >  = 3 or <  = − 3 (~ 9 × linear change). The resulting p-values were corrected for multiplicity by applying Benjamini–Hochberg adjustment to all p-values calculated for a time point with a false discovery rate (FDR) < 5%. Genes with an adjusted *p* value < 0.05 were considered significant. The STRING database was used to show protein–protein interactions (string-db.org).

### RT-PCR

Total RNA was extracted (ExtractMe) and exposed to reverse transcription (SensiFAST™, Bioline Reagents Ltd., London, UK). RT-PCR was done according to the manufacturer’s instructions (LabQ master mix; LabConsulting, Vösendorf, Austria) on a CFX Connect PCR device (BioRad, Hercules, CA, USA). Primer sequences are given in Table 1. Calculation of relative gene expression was based on delta delta CT method using a software (CFX Maestro™, BioRad). Reactions were done in duplicates.

**Table1:** Primer sequence

	Sequence F	Sequence R
hGAPDH	aagccacatcgctcagacac	gcccaatacgaccaaatcc
hβactin	ccaaccgcgagaagatga	ccagaggcgtacagggatag
hCXCL13	ctctgcttctcatgctgctg	gctctcttggacacatctacacc
hKANK4	gctgctgtcggcctactc	gatttaaggctggtcgatgg
hJPH3	acggggccaaatacgaag	cactggccctggtaggtc
hOPCML	ccttgtacccacaggagtgc	ggttacccggtcatctatgg
hST6GAL2	cagcccaacttcccagtg	tcagcaccttgtgtcttaatgc
hKRT16	ctattcttcccgcgaggtc	gaagctggatgagctctgct
hIL11	aaataaggcacagatgcc	ccttccaaagccagatc

### Western Blot analysis

Gingival fibroblasts were serum-starved overnight and then preincubated for 30 min with 5% human milk. Cell extracts containing SDS buffer and protease inhibitors (PhosSTOP with cOmplete; Sigma, St. Louis, MO) were separated by SDS-PAGE and transferred onto nitrocellulose membranes (Whatman, GE Healthcare, General Electric Company, Fairfield, CT). Membranes were blocked and the binding of the first antibody raised against psmad-3 (rabbit; phospho S423 + S425; EP823Y, Abcam, Cambridge, UK) and the Smad-3 (mouse; Smad-3 (38-Q): sc-101154, Santa Cruz Biotechnology, SCBT, Santa Cruz, CA, USA), was detected with the appropriate secondary antibody linked to a peroxidase. Chemiluminescence signals were visualized with the ChemiDoc imaging system (Bio-Rad Laboratories, Inc., Hercules, CA).

### Immunofluorescence

Gingival fibroblasts exposed to human milk for 24 h were incubated with anti-Smad2/3 antibody (D7G7 XP® Rabbit mAb, Cell Signalling Danvers, MA) and for overnight at 4 °C. Following blocking by 1% BSA and permeabilization with 0.1% Triton X, an Alexa Fluor® 488-conjugated secondary antibody (Cell Signalling) was added for 1 h at room temperature. Images were captured under a fluorescent microscope (Axio Imager M2, Carl Zeiss AG, Oberkochen, Germany).

### Statistical analysis

All experiments were repeated at least three times. Data from individual experiments are shown as dot-blots. Statistical analysis was based on Paired t test (Fig. 3, 4, 5, and 8). Data were analyzed by the Prism 8.0e software (GraphPad Software; San Diego, CA). The p-values are indicated in the respective figures.

## Supplementary Information

**Additional file 1: Table S1.** RNA sequencing of gingival fibroblasts exposed to milk revealed a total number of 142 genes being at least nine fold up- and down-regulated by milk and 99 genes, were blocked by the inhibitor for the TGF-β receptor typeI kinase.

## Data Availability

The datasets used and/or analyzed during the current study are available from the corresponding author on reasonable request.
